# A Novel Method to Determine the Respiratory Compensation Point from Percutaneous Oxygen Saturation of Healthy Adults During a Ramp-Incremental Test: A Cross-Sectional Study

**DOI:** 10.3390/medsci13030192

**Published:** 2025-09-15

**Authors:** Masatsugu Abe, Kai Ushio, Masaya Tsubokawa, Koki Fukuhara, Yoshitaka Iwamoto, Daisuke Iwaki, Yuki Nakashima, Takeshi Nakamura, Yukio Mikami

**Affiliations:** 1 FANCL New Business Head Office, 12-13 Kamishinano, Totsuka-ku, Yokohama 244-0806, Japan; abe_masatsugu@fancl.co.jp; 2 Department of Rehabilitation Medicine, Hiroshima University Hospital, 1-2-3 Kasumi, Minami-ku, Hiroshima 734-8551, Japan; mikamiy@hiroshima-u.ac.jp; 3 Department of Rehabilitation Medicine, Yokohama City University School of Medicine, 3-9 Fukuura, Kanazawa-ku, Yokohama 236-0004, Japan; take_n@yokohama-cu.ac.jp; 4 FANCL Corporation Research Institute, 12-13 Kamishinano, Totsuka-ku, Yokohama 244-0806, Japan; tsubokawa_masaya@fancl.co.jp; 5 Division of Rehabilitation, Department of Clinical Practice and Support, Hiroshima University Hospital, 1-2-3 Kasumi, Minami-ku, Hiroshima 734-8551, Japan; kouki@hiroshima-u.ac.jp (K.F.); iwamo10@hiroshima-u.ac.jp (Y.I.); dai-iwaki@hiroshima-u.ac.jp (D.I.); ynakashi@hiroshima-u.ac.jp (Y.N.)

**Keywords:** ventilatory threshold 1, cardiopulmonary exercise testing, percutaneous oxygen saturation, pulse oximeter, ventilatory threshold 2

## Abstract

**Background:** In exercise testing, the ventilatory threshold 1 (VT1) and ventilatory threshold 2 (VT2) are used in lifestyle-related diseases, cardiac rehabilitation, and athletic training. We investigated a VT2 measuring method using a pulse oximeter. **Methods:** Thirty-four adults (men: 15; women: 19) performed a bicycle ergometer Ramp Test. VT1 values were determined using expiratory gas data. The bifurcation of the curve obtained by designating the pulse rate (PR) as an independent variable and SpO_2_/PR as a dependent variable was calculated using the residual sum of squares and defined as the SpO_2_ threshold (ST) (SpO_2_-Slope method). A second bifurcation with ST as the origin was further defined (ST2). ST2 validity was assessed by comparing and analyzing the differences and correlations with each VT2 obtained by expiratory gas analysis. **Results:** The correlation between ST2 determined by the SpO_2_-Slope method using PR as an index and VT2 obtained from respiratory gas analysis was significant, showing a positive correlation (r = 0.74~0.92; *p* < 0.01), with most data points falling within the 1.96 ± SD in the Bland–Altman analysis. **Conclusions:** ST2 values derived from SpO_2_ and pulse rate measurements by pulse oximeter may be a valuable VT2 measuring method.

## 1. Introduction

When conducting exercise challenge testing, choosing the appropriate exercise intensity is essential for safe and effective rehabilitation and improving athletic performance. The achievement of a certain exercise intensity threshold has also been used to assess the response to treatment and predict the prognosis. Standards for the cardiopulmonary exercise capacity in cardiopulmonary exercise testing (CPET) include the ventilatory threshold 1 (VT1) [1], the transition point where the energy supply switches from the aerobic metabolism to anaerobic metabolism when exercise is started at an easy level, and its intensity is gradually increased; and the respiratory ventilatory threshold 2 (VT2) [2,3], where the maximum workload that can be maintained before severe hyperventilation due to metabolic acidosis occurs. A joint statement from Europe, the United States, and Canada provides evidence-based proposals to move from ‘range-based’ to ‘threshold-based’ aerobic exercise intensity prescriptions [4]. The European Society of Preventive Cardiology’s position statement states that the evaluation of VT1 and VT2 during CPET provides reliable and effortless parameters that should be used to determine the intensity of aerobic exercise for the majority of patients who have cardiovascular diseases (CVD) [5]. Heart disease patients who present with the identification of not only VT1 but also VT2 during CPET reduce their risk of left ventricular assist device implantation, heart transplant, and cardiovascular death [6]. In addition, patients with heart disease who reach the VT2 during CPET reportedly have a higher peak oxygen intake (peak VO_2_ max) and a higher VT1 during peak exercise [7]. This makes the measurement of the VT2 in patients with heart disease extremely important.

Expiratory gas analysis during CPET is the gold standard technique for VT1 and VT2 measurements. Although this is a non-invasive method that is widely used in the fields of sports and medicine, it needs high-priced equipment and specialists to measure the VT1 and VT2 [8,9]. A number of alternative devices for non-invasive measurement of the VT1 and VT2 in incremental exercise instead of expiratory gas analyzers are currently under development. One method being tested is the detection of VT1 in healthy individuals and patients who have CVD by measuring the lactic acid concentration in sweat [10,11]. A method for detecting the VT1 and VT2 from muscle activity, measured using electromyography (EMG), has also been shown to be effective in healthy individuals, patients with chronic illness, and patients with COVID-19 [12,13,14,15].

Against this backdrop, we developed a method for automatically calculating VT1 from pulse rate (PR) and percutaneous oxygen saturation (SpO_2_) measurements made by a pulse oximeter, which is commonly used in daily medical practice, and confirmed its correlation and concordance with the VT1 obtained with an expiratory gas analysis [16]. This is based on the background that the SpO_2_ decreased around VT1 during incremental exercise [17], but SpO_2_ has also been reported to show a decrease before VT2 as well as VT1 [18]. Therefore, we hypothesized that our pulse oximeter-based method could estimate not only VT1 but also VT2. This study aimed to verify the correlation between VT2 derived from SpO_2_ and PR data using a pulse oximeter and VT2 determined by breath gas analysis.

## 2. Materials and Methods

### 2.1. Study Design, Ethics Approval, and Consent to Participate

This investigation, designed as a cross-sectional study, was reviewed and approved by the Clinical Research Ethics Committee of FANCL Corporation (Yokohama, Japan, Approval No. C2022-13; 28 September 2022). All study procedures were conducted in accordance with the Helsinki Declaration and complied with the Ethical Guidelines for Medical and Human-Participants Related Biological Research. Written informed consent was obtained from every participant prior to their inclusion in the cross-sectional study. In addition, the study was formally registered in the UMIN Clinical Trials Registry (UMIN000049277).

### 2.2. Sample Size Calculation and Eligibility Criteria

Sample size calculation assumed no difference between the two measurement methods and a standard deviation of the relative error rate of 30%; 26 participants were required to ensure 90% statistical power so that the 95% confidence interval of the relative error rate would be within the range of −20% to +20%. Furthermore, considering dropouts, at least 30 participants were required.

A total of 19 male and 15 female participants entered via the FANCL Corporation website. The inclusion requirements were as follows: (1) men and women in good health who were 20 to 64 years old when giving consent and (2) BMI (body mass index) between 18.5 kg/m^2^ and under 30 kg/m^2^. The criteria for exclusion were defined as follows: (1) the presence of severe respiratory, hepatic, gastrointestinal, renal, or cardiac disease; (2) unwillingness to undergo a high-intensity exercise test; (3) planning pregnancy during the study, pregnant at present (suspected cases included), or breastfeeding at the time; (4) having an active or recent record of COVID-19 infection or having previously been in close contact with an individual diagnosed with the illness; (5) any condition triggered by exercise, such as arrhythmia, anaphylaxis, or motor dysfunction; and (6) any other reason deemed by the investigator to make participation inappropriate.

### 2.3. Experimental Methods

After providing informed consent, participants were requested to provide their birth date, age (at the time of consent), any current medical conditions (including ongoing treatments and medications), physical activity routines, current smoking condition, and alcohol intake. Before the test, participants were advised to refrain from overeating or heavy drinking the previous day, to obtain adequate sleep the night before, and to consume only water and a specified food on the day of testing [16]. Calorie Mate Jelly, flavored with lime and grapefruit, was selected as the specified food (Otsuka Pharmaceutical, Tokyo, Japan), providing 200 kcal and comprising 0.11 g of salt equivalent, 8.2 g of protein, 33.2 g of carbohydrates, and 4.4 g of fat. The test was conducted in the morning under controlled laboratory conditions at a room temperature of 22 °C [16].

### 2.4. Exercise Test Method

The exercise test was conducted using a ramp protocol on an ergometer (Corival Cpet, Kyokko Busan, Tokyo, Japan), with the saddle adjusted to maintain a slight bend in the participants’ knees at the lowest point of the pedal stroke [16]. Following a 2 min rest period, participants completed a 5 min warm-up at 20 watts and 60 revolutions per minute (rpm) [16]. Subsequently, the exercise load was gradually increased in a Ramp Test at a rate of 1 watt every 6 s until pre-determined termination criteria were reached [16]. These criteria included the following: (1) unable to maintain a pedaling rate of 60 rpm as a result of leg fatigue; (2) attaining a heart rate exceeding 85% of the maximum predicted for age (estimated as 220 minus the individual’s age); or (3) the investigator’s decision to terminate the test [16].

### 2.5. Expiratory Gas Analysis

Measurements of carbon dioxide output (VCO_2_), oxygen uptake (VO_2_), respiratory exchange ratio (R = VCO_2_/VO_2_), and minute ventilation (VE) were obtained through an expiratory gas analysis system (AE300S, Minato Medical Science, Tokyo, Japan). Expired gases collected via a mixing chamber method were analyzed to measure carbon dioxide output (VE/VCO_2_), ventilatory equivalents for oxygen (VE/VO_2_), end-tidal carbon dioxide concentration (PETCO_2_), and end-tidal oxygen (PETO_2_). The participants’ HR was monitored continuously using a chest-mounted sensor (POLAR Heart Rate Sensor T31 N, manufactured in Polar Electro Inc., Tokyo, Japan). At the same time, SpO_2_ and PR were recorded with a pulse oximeter equipped with a finger probe (DS100A Nelcor Sensor, Covidien Inc., Tokyo, Japan) placed on the participant’s left index finger [16]. Exhaled gas metrics and HR were documented at 20 s intervals, whereas SpO_2_ and PR were logged every 4 s. For the purpose of analysis, data collection was initiated during the final minute of the warm-up period and was maintained through to the conclusion of the exercise assessment.

### 2.6. Calculation of the RP Point by Doctors

VT2 was determined daily by the physicians involved in CPET using expiratory gas analysis. The VT2 was determined using three different methods: the VE/VCO_2_ method (the point at or above the VT1 at which VE/VCO_2_ started to consistently increase), the PETCO_2_ method (the point at or above the VT1 at which PETCO_2_ started to consistently decrease), and the VE_VCO_2_-Slope method (the point at or above the VT1 at which the rate of increase in VE started to outstrip the rate of increase in VCO_2_). The VO_2_ and load associated with the PR at the VT2 were identified.

### 2.7. Automated Calculation of the ST2 Using SpO_2_

A secondary regression analysis was performed using pulse oximetry data, specifically SpO_2_ and pulse rate (PR). In this analysis, PR was used as the predictor variable, while the ratio of SpO_2_ to PR was treated as the response variable. The turning point of the regression curve was identified at the position that minimized the residual sum of squares, and this point was defined as the SpO_2_ threshold (ST), according to the SpO_2_-Slope method. Next, the SpO_2_-Slope method was applied again, this time starting from ST as the new baseline, and the following point of curve intersection was defined as the second SpO_2_ threshold (ST2). The specific procedure for determining ST2 is illustrated in Figure 2a. During CPET, to minimize artifact noise when measuring SpO_2_ and PR with a pulse oximeter, participants were instructed to keep their hands fixed on the handle and to avoid gripping their fingers tightly. The simple ST2 calculation method is described in Supplementary File S1.

### 2.8. Statistical Analysis

We investigated the differences, error rates, and correlations between the PR, VO_2_, and load at the VT2, determined by three different methods of expiratory gas analysis (VE/VCO_2_, PETCO_2_, and VE-VCO_2_-Slope) and the PR, VO_2_, and load at the VT2, determined from pulse oximetry data. Only actual data were analyzed, with no imputation of indeterminate missing values. The variability between the two measurements was assessed by determining the 95% confidence interval (CI 95%) of the mean difference. If the 95% confidence interval (CI) included zero, this suggested that the difference observed between the two measurements lacked statistical significance. The error between the two measured values was calculated by dividing the VT2, determined by SpO_2_, by the VT2 determined by expiratory gas analysis. The proportion of the data included in the tolerance limits (within the mean difference ±1.96 SD) from the Brand–Altman analysis was calculated. The relationship between the two measured values was assessed by computing Pearson’s correlation coefficient (r). Correlation coefficients with absolute values between 0.4 and 0.7 were classified as moderate, whereas those ranging from above 0.7 up to 1.0 were deemed strong. We performed statistical analyses using JMP^®^ 17.0 software (SAS Institute Japan Ltd., Tokyo, Japan).

## 3. Results

**Figure 1:**
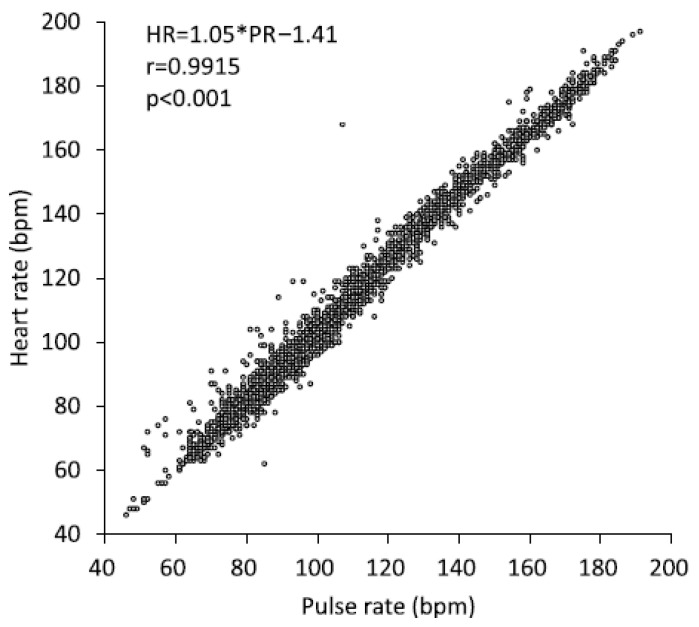
The relationship between PR and HR was examined. Data were gathered starting at the resting phase and continuing throughout the full loading stage. PR measured with a pulse oximeter, whereas HR was recorded using a heart rate monitor. To evaluate the strength of the association, Pearson’s correlation coefficient (r), along with the corresponding *p*-value, was calculated. HR: heart rate, PR: pulse rate.

**Figure 2:**
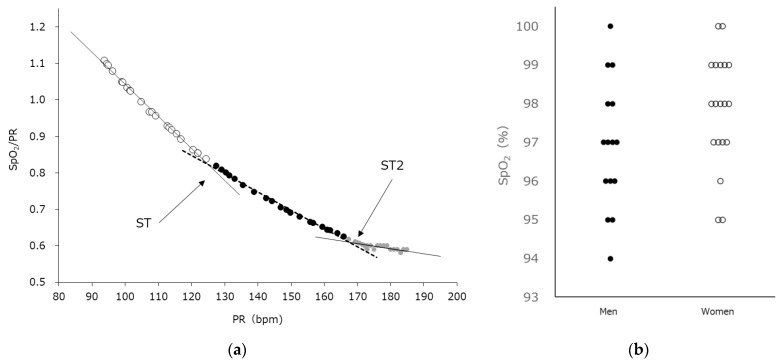
(**a**) SpO_2_-Slope method. ST denotes the point at which the total sum of squared residuals from two separate regression lines reaches its lowest value. Starting from ST, ST2 is determined as the point at which this sum of squared residuals attains its minimum once more. (**b**) The range of SpO_2_ values associated with ST2. SpO_2_ varied from 94% up to 100%. HR: heart rate, PR: pulse rate, SpO2: saturation of peripheral oxygen, ST: percutaneous oxygen saturation threshold, and ST2: percutaneous oxygen saturation threshold 2.

Table 1 provides a detailed summary of the participants’ characteristics. The mean body mass index (BMI) was 22.1 ± 0.4 kg/m^2^, varying from 18.3 to 27.9 kg/m^2^, and the mean age was 41.2 ± 1.6 years (range: 27–63 years). Figure 1 presents a scatter plot showing the relationship between the PR and HR for each participant. A strong relationship was observed between the PR and HR at rest during CPET (r = 0.995, *p* < 0.001). Beyond a specific time during the CPET, both SpO_2_ and PR began to decline as exercise intensity escalated. Figure 2a shows the representative results of the SpO_2_-Slope method. Figure 2b shows the SpO_2_ values of all participants at ST2.

In Table 2, the PR, VO_2_, and workload values are shown for the VT2 identified using the three different measurement approaches, along with those recorded at ST2 (mean ± standard deviation). Some VT2 data were missing due to difficulties for the physicians to identify values and were excluded from the analysis. The ultimate analysis comprised 31 participants for both the VE/VCO_2_ and PETCO_2_ methods and 32 participants for the VE_VCO_2_-Slope method. The mean PR was 162.3 ± 15.8 with the VE/VCO_2_ method, 160.3 ± 15.7 with the PETCO_2_ method, and 168.9 ± 15.3 with the VE_VCO_2_-Slope method, and at ST2, it was 153.4 ± 15.2. VO_2_ was 1747.1 ± 577.5 with the VE/VCO_2_ method, 1660.5 ± 535.8 with the PETCO_2_ method, and 1849.7 ± 594.7 with the VE_VCO_2_-Slope method, and at ST2, it was 1632.9 ± 481.8. The load was 140.0 ± 42.4 with the VE/VCO_2_ method, 132.9 ± 34.8 with the PETCO_2_ method, and 148.8 ± 42.3 with the VE_VCO_2_-Slope method, and at ST2, it was 132.6 ± 36.0. Each parameter was higher in men than in women.

Table 3 presents the differences between the values at the VT2 obtained by the three measurement methods and those at ST2. The mean differences (95% CI upper limit/lower limit) between PR at ST2 and PR measured by the VE/VCO_2_, PETO_2_, and VE_VCO_2_-Slope methods were −8.6 (95%CI; −12.8 to −4.54), −6.7 (95%CI; −10.8/−2.6), and −15.1 (95%CI; −19.1 to −11.1), respectively. Because the 95% CI did not include zero, it was considered that there were differences between the two measured values. For the VO_2_ and load values, the only mean difference for which the 95% CI did not include zero was with the value in the VE_VCO_2_-Slope method; it was determined that there were only differences between the two values with the VE_VCO_2_-Slope method. These results were similar in the gender subgroup analysis.

Table 4 presents the correlations between the values at the VT2 obtained by the three measurement methods and those at ST2.The correlation coefficients between the PR at ST2 and its value measured by the VE/VCO_2_, PETCO_2_, and VE_VCO_2_-Slope methods yielded correlation coefficients of 0.74, 0.75, and 0.74, respectively (all *p* < 0.01), indicating a significant positive association between the paired measurements. Comparable robust positive correlations were also found between the VO_2_ and load measurements. In men, no significant correlation was found between the pulse rate of VE/VCO_2_ and PETCO_2_ and ST2, but a significant correlation was observed in all other items in the gender subgroup analysis. The correlation plot and the corresponding linear regression lines are presented in Supplementary File S2.

**Figure 3:**
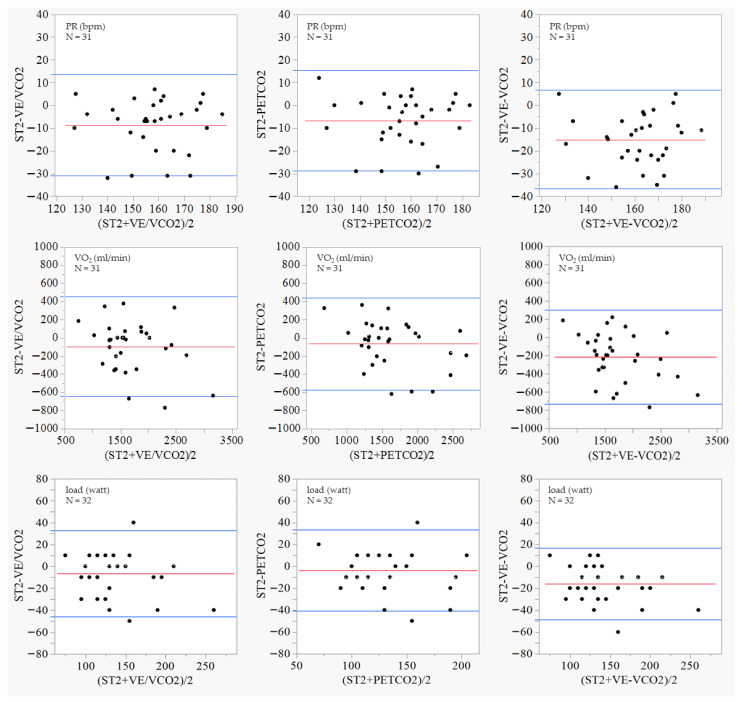
Bland–Altman plot analyses. PETCO_2_: end-tidal oxygen concentration, ST2: percutaneous oxygen saturation threshold 2, VCO_2_: carbon dioxide, VE: ventilation, and VE/VCO_2_: oxygen ventilatory equivalent. The red line represents the mean difference between the two measurement methods, and the blue lines indicate ±1.96 SD.

Figure 3 shows the Bland–Altman plot analyses of the PR (bpm), VO_2_ (ml/min), and load (watt) at the ST2, VE/VCO_2_, PETCO_2_, and VE–VCO_2_ Slope. While most of the plots were distributed within ±1.96 SD, the values of PR, VO_2_, and the load at ST2 were consistently lower compared with those at the VE/VCO_2_, PETCO_2_, and VE–VCO_2_ Slope. The proportions for which the absolute error between the PR at ST2 and its value measured by the VE/VCO_2_, PETCO_2_, and VE/VCO_2_ methods were included in the tolerance limits (within the mean difference ± 1.96 SD) and were 87.1%, 90.3%, and 100.0%. Similarly, for the VO_2_ and load values, it was confirmed that the proportions for which the absolute error was within, including the tolerance limits of the two measurement methods, was high in all these cases.

## 4. Discussion

We investigated whether ST2, the second bifurcation automatically calculated from SpO_2_ and PR data measured using a pulse oximeter, is a viable alternative to VT2 measurement using conventional expiratory gas analysis in incremental exercise in this study. Our results showed that, although the PR was lower at ST2 than that at the VT2 obtained by the expiratory gas analyzer, it exhibited a correlation. VO_2_ and the load also exhibited similarly high correlations with the PR. Accordingly, we demonstrated the effectiveness of the SpO_2_-Slope method for accurately identifying the VT2 from the SpO_2_ and PR parameters.

Increased body temperature and lactic acidosis, in line with increased exercise intensity, reduced the arterial oxygenated hemoglobin [19,20,21]. Previous studies using an arterial blood gas analysis have consistently demonstrated that arterial oxygen tension declines with progressively increasing exercise intensity [22,23]. In addition to arterial oxygen pressure, arterial oxygen saturation (SaO_2_), bicarbonate ions, and arterial oxygenated hemoglobin all decrease during incremental exercise [19,20,21], and SpO_2_, which is measured by pulse oximeter and demonstrates arterial oxygen saturation, has also been shown to decrease during gradual increase exercise [24]. Based on these findings, it was shown that the inflection points where SpO_2_ expeditiously decreased occurred at the load equivalent to the VT1 [17]. It was also shown that during incremental exercise, SpO_2_ decreased before VT1 and VT2 and that the time until the second drop in SpO_2_ had a strong correlation with the time until VT1 [18]. The fact that the SpO_2_ decreased before the VT2 could be consistent with that the PR at ST2 was lower than the PR at VT2 in this study. They also found that the time from the start of exercise to the second drop in SpO_2_ was moderately correlated with the VO_2_ max [18], suggesting that it may be possible not only to calculate the VT1 and the VT2 but also to predict the VO_2_ max from the drop in SpO_2_.

Accordingly, PaO_2_, SaO_2_, and SpO_2_ are believed to decrease as exercise intensity increases during incremental exercise. However, this behavior is not simple [18,22,23,24]. Therefore, the inflection point at which the SpO_2_ begins to decrease is difficult to determine accurately by visual inspection. In a previous study, we reported that ST calculated using the SpO_2_-Slope method approximates AT [16]. Because estimation of the AT and VT2 by expiratory gas analysis is a human-based technique, it may not always be possible to identify the VT1 and VT2, but ST and ST2 can be calculated automatically by the SpO2-Slope method, meaning that the appearance of the VT1 and VT2 can be confirmed objectively. The participants in this study were healthy adults who were expected to reach the VT2; however, this point was not identified by the conventional VE/VCO_2_ and PETCO_2_ methods in three cases and by the VE_VCO_2_-Slope method in two. Nonetheless, it was successfully determined using the SpO_2_-Slope method in all participants. If it can be confirmed in the future that the appearance of VT1 and VT2 can also be accurately determined in heart disease patients using the SpO_2_-Slope method, this could be useful in the rehabilitation, treatment, and prediction of the prognosis of patients with heart disease.

Several alternative devices for the non-invasive measurement of VT1 and VT2 in incremental exercise instead of expiratory gas analyzers are currently under development. A method under investigation involves identifying VT1 in both healthy participants and patients with cardiovascular disorders through the assessment of lactic acid levels in sweat [10,11]. The lactate threshold (LT) in sweat (sLT), measured using an LT sensor, has been shown to have a high correlation and concordance with the LT in blood (bLT) and the ventilatory threshold during incremental exercise [10]. In addition, sLT and bLT have been shown to be correlated and exhibit concordance, even in healthy men who start to sweat quickly [11]. A method for detecting VT1 and VT2 from muscle activity measured by EMG has also been shown to be effective in healthy individuals and those with illnesses [12,13,14,15]. When the activity of the lateral vastus muscle measured by EMG during an incremental pedaling exercise was evaluated using the root mean square (RMS) method, correlation and concordance were demonstrated between the first and second EMG-elevated breaking points and the VT1 and VT2 measured by VE/VO_2_ [15]. Near-infrared spectroscopy (NIRS) measurements of the brain blood flow during incremental exercise also showed that the total hemoglobin level in the frontal cortex (prefrontal cortex, premotor area, and supplementary motor area) increased significantly at the VT2 and at the point of peak oxygen consumption compared with that at rest [15]. As none of these methods require an expiratory gas mask, they should greatly reduce the discomfort experienced by the participants during measurement. Our SpO_2_-Slope method also holds similar promise, and as it uses a pulse oximeter, which is an essential medical device in everyday clinical practice, widespread device availability is another strength.

Telerehabilitation is the method of using devices such as smartphones, tablets, and personal computers to provide patients with rehabilitation at home or elsewhere with no physical contact. It has attracted widespread attention due to the COVID-19 outbreak, and its effectiveness in terms of physical function, quality of life (QOL), cost-effectiveness, and other advantages have been demonstrated in studies on patients with conditions including cerebral stroke [25,26], proximal femoral fracture [27], and cancer [28]. Studies on home-based telerehabilitation for cardiac rehabilitation have also been conducted, and these have demonstrated its effects on short- and long-term physical function and cost-effectiveness [29,30,31,32,33,34]. In such telerehabilitation, wearable devices for the real-time measurement of vital signs and the assessment of activity levels play an essential role in safety management and in confirming that the rehabilitation is actually being carried out. Among these, the SpO_2_ measurement is considered vital [31,33,34]. In this situation, guidance on the amount of aerobic exercise is given according to the Borg scale [31], with the level starting at 40–60% of the ventilation threshold 1(VT1) during CPET or peak VO_2_ and gradually increasing according to the rate of perceived exercise (RPE) [33] and decreasing in the event of increases in parameters such as weight, heart rate, or heart failure marker levels in blood tests [34]. An accelerometer was used to monitor and adjust the exercise program and daily activity levels [32]. The development of pulse oximeters with Bluetooth connectivity as wearable devices is already underway, and their introduction into telerehabilitation will not only be useful for safety monitoring and management during rehabilitation, but also have the potential to enable the appropriate and non-invasive adjustment of the exercise load without the use of an expiratory gas analysis. This may contribute to its wider adoption and promotion in the future as a device that is not limited solely to safety monitoring in telerehabilitation.

This study has several limitations. The participants were only healthy Japanese people. The results may not apply to athletes or patients. Skin pigmentation also may cause racial differences in the pulse oximeter values [35]. The test meal was administered prior to the clinical assessments. This may have influenced exhaled gas metabolism. Some participants exceeded the recommended exercise duration of 8–12 min. This may have led to an overestimation of VT2 [36,37]. A cycle ergometer was used as an exercise method. It remains unclear whether similar results would be obtained with treadmill or step exercises. A mixing chamber method was used to accurately evaluate the respiratory metabolic function. This method may have reduced the temporal resolution and underestimated the variability. The ST2 was associated with lower HR and VO_2_ values compared to the conventional VT2. This point should be kept in mind when applying it to clinical practice or training. Finaly, this study was a cross-sectional study. Causal relationships cannot be established. Further long-term research is necessary, taking these limitations into account.

## 5. Conclusions

This is the first study to report a strong correlation and low relative error rate between ST2 (the second point at which SpO_2_ abruptly decreases), calculated from SpO_2_ and PR, using the SpO_2_-Slope method and the VT2 demonstrated by the respiratory gas method during incremental exercise. Based on the results of this study conducted in healthy adults, this finding suggests that the SpO_2_-Slope method can be a valid approach for the highly accurate and simple measurement of VT2. Taken together with our previous study, these results suggest that VT1 and VT2 can be measured simply and accurately by this method. This approach may reduce both the burden and cost for patients. In addition, it has the potential to improve endurance, extend healthy longevity, enhance athletic competitiveness, strengthen safety management, and support the wider use of telerehabilitation in exercise therapy. However, as this study is preliminary, further clinical trials are warranted in the future.

## Figures and Tables

**Table 1:** Participant characteristics.

Characteristics	Unit	All (*N* = 34)	Men (*N* = 15)	Women (*N* = 19)
Age	Years	41.2 ± 9.4	36.7 ± 10.2	44.8 ± 10.2
Height	Cm	164.9 ± 8.3	171.3 ± 6.4	159.7 ± 6.4
Weight	Kg	60.2 ± 9.4	65.6 ± 8.4	55.8 ± 8.4
BMI	kg/m^2^	22.1 ± 2.5	22.3 ± 2.4	21.9 ± 2.4

Values are shown as mean  ±  standard deviation. BMI: body mass index.

**Table 2:** PR, VO_2_, and load at each VT2 and ST2.

Gender	Characteristics	N	Pulse Rate (bpm)	VO_2_ (mL/min)	Load (watts)
			Mean ± SD	Mean ± SD	Mean ± SD
All	VE/VCO_2_	31	162.3 ± 15.8	1747.1 ± 577.5	140.0 ± 42.4
	PETCO_2_	31	160.3 ± 15.7	1660.5 ± 535.8	132.9 ± 34.8
	VE-VCO_2_-Slope	32	168.9 ± 15.3	1849.7 ± 594.7	148.8 ± 42.3
	SpO_2_-Slope (ST2)	34	153.4 ± 15.2	1632.9 ± 481.8	132.6 ± 36.0
Men	VE/VCO_2_	13	166.2 ± 17.8	2188.5 ± 571.6	170.8 ± 46.6
	PETCO_2_	12	163.8 ± 18.3	2097.5 ± 514.1	161.7 ± 34.3
	VE-VCO_2_-Slope	13	173.4 ± 17.2	2333.6 ± 568.7	183.1 ± 42.3
	SpO_2_-Slope (ST2)	15	153.9 ± 15.4	1948.5 ± 492.7	156.7 ± 37.7
Women	VE/VCO_2_	18	159.5 ± 12.0	1428.2 ± 318.2	117.8 ± 19.9
	PETCO_2_	19	158.1 ± 10.0	1384.6 ± 330.6	114.7 ± 19.8
	VE-VCO_2_-Slope	19	165.9 ± 11.1	1518.6 ± 329.6	125.3 ± 21.4
	SpO_2_-Slope (ST2)	19	153.1 ± 15.6	1383.8 ± 297.0	113.7 ± 20.3

Values are shown as mean  ±  standard deviation. bpm: beats per minute, PETCO_2_: end-tidal oxygen concentration, ST2: percutaneous oxygen saturation threshold 2, VCO_2_: carbon dioxide, VE: ventilation, VO_2_: oxygen consumption, and VE/VCO_2_: oxygen ventilatory equivalent.

**Table 3:** Agreement, concordance, and association between VT2 and ST2.

Gender	Characteristics	N	Pulse Rate (bpm)		VO_2_ (mL/min)		Load (watts)	
			Diff	95% CI	Diff	95% CI	Diff	95% CI
All	VE/VCO_2_	31	−8.6	−12.8 to −4.4	−98.6	−200.8 to 3.5	−6.5	−13.8 to 0.9
	PETCO_2_	31	−6.7	−10.8 to −2.6	−66.2	−161.1 to 28.8	−3.5	−10.5 to 3.4
	VE-VCO_2_-Slope	32	−15.1	−19.1 to −11.1	−216.3	−311.8 to −120.8	−15.9	−21.9 to −9.9
Men	VE/VCO_2_	13	−11.2	−19.7 to −2.7	−190.4	−411.1 to 30.3	−10.0	−26.2 to 6.2
	PETCO_2_	12	−9.3	−18.1 to −0.6	−169.8	−379.9 to 40.4	−7.5	−24.7 to 9.7
	VE-VCO_2_-Slope	13	−18.4	−25.1 to −11.6	−335.5	−527.6 to −143.4	−22.3	−33.9 to −10.7
Women	VE/VCO_2_	18	−6.7	−11.1 to 2.2	−32.4	−120.8 to 56.0	−3.9	−10.7 to 3.0
	PETCO_2_	19	−5.0	−9.4 to −0.6	−0.7	−88.6 to 87.1	−1.1	−6.6 to 4.5
	VE-VCO_2_-Slope	19	−12.8	−18.0 to −7.6	−134.7	−226.5 to −43.0	−11.6	−18.1 to −5.1

Values are shown as mean  ±  standard deviation. bpm: beats per minute, PETCO_2_: end-tidal oxygen concentration, ST2: percutaneous oxygen saturation threshold 2, VCO_2_: carbon dioxide, VE: ventilation, VO_2_: oxygen consumption, and VE/VCO_2_: oxygen ventilatory equivalent.

**Table 4:** Correlation between VT2 and ST2.

Gender	Characteristics	N	Pulse Rate (bpm)		VO_2_ (mL/min)		Load (watts)	
			r	* p *	r	* p *	r	* p *
All	VE/VCO_2_	31	0.74	<0.01	0.88	<0.01	0.88	<0.01
	PETCO_2_	31	0.75	<0.01	0.88	<0.01	0.84	<0.01
	VE-VCO_2_-Slope	32	0.74	<0.01	0.90	<0.01	0.92	<0.01
Men	VE/VCO_2_	13	0.54	0.057	0.78	<0.01	0.82	<0.01
	PETCO_2_	12	0.58	0.048	0.77	<0.01	0.67	0.017
	VE-VCO_2_-Slope	13	0.73	<0.01	0.83	<0.01	0.89	<0.01
Women	VE/VCO_2_	18	0.86	<0.01	0.84	<0.01	0.77	<0.01
	PETCO_2_	19	0.87	<0.01	0.84	<0.01	0.84	<0.01
	VE-VCO_2_-Slope	19	0.79	<0.01	0.82	<0.01	0.79	<0.01

Values are shown as mean  ±  standard deviation. bpm: beats per minute, PETCO_2_: end-tidal oxygen concentration, ST2: percutaneous oxygen saturation threshold 2, VCO_2_: carbon dioxide, VE: ventilation, VO_2_: oxygen consumption, and VE/VCO_2_: oxygen ventilatory equivalent.

## Data Availability

The original contributions presented in this study are included in the article/Supplementary Materials. Further inquiries can be directed to the corresponding authors.

## Supplementary Materials

The following supporting information can be downloaded at: https://www.mdpi.com/article/10.3390/medsci13030192/s1, Supplementary File S1: ST2 calculation flowchart. Supplementary File S2: Each VT2 and ST2_Bland-Altman plot.

## Abbreviations

This manuscript employs the following abbreviations:

VT1	ventilatory threshold 1
VT2	ventilatory threshold 2
ST	SpO_2_ threshold
CPET	cardiopulmonary exercise testing
VT	ventilatory threshold
CVD	cardiovascular diseases
peak VO_2max_	peak oxygen intake
EMG	electromyography
SpO_2_	oxygen saturation
PR	pulse rate
BMI	body mass index
VO_2_	oxygen consumption
VCO_2_	carbon dioxide production
VE	minute ventilation
VE/VO_2_	ventilatory equivalents for oxygen
VE/VCO_2_	ventilatory equivalent for carbon dioxide
PETO_2_	end-tidal oxygen concentration
PETCO_2_	end-tidal carbon dioxide concentration
HR	heart rate
ST2	second SpO_2_ threshold
CI	confidence interval
LT	lactate threshold
sLT	LT in sweat
bLT	LT in blood
NIRS	near-infrared spectroscopy
QOL	quality of life
RPE	rate of perceived exercise
