# Supplementary material for: A Novel Method to Determine the Respiratory Compensation Point from Percutaneous Oxygen Saturation of Healthy Adults During a Ramp-Incremental Test: A Cross-Sectional Study
Source: Med Sci (Basel). 2025 Sep 15;13(3):192. doi: 10.3390/medsci13030192 (PMC12452715; doi:10.3390/medsci13030192)

S1. ST2 calculation flowchart.

1, Perform SpO_2/PR measurements and generate a scatter plot forming an arc.

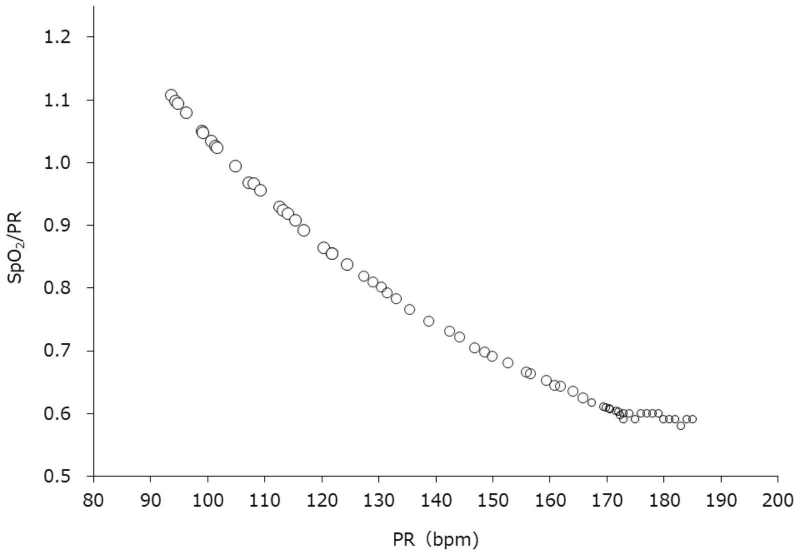

2, Apply the least squares method to the entire arc-shaped plot obtained in step 1 to calculate ST.

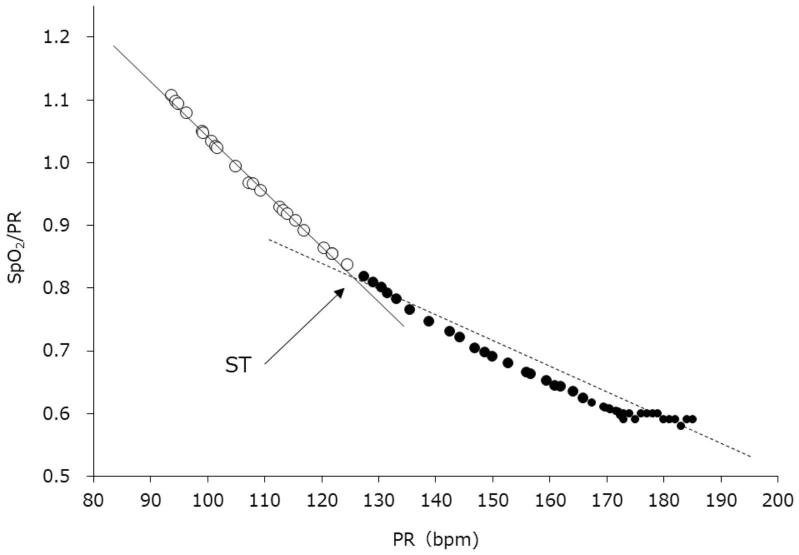

3, Using the ST calculated in step 2 as the starting point, apply the least squares method again to calculate ST2.

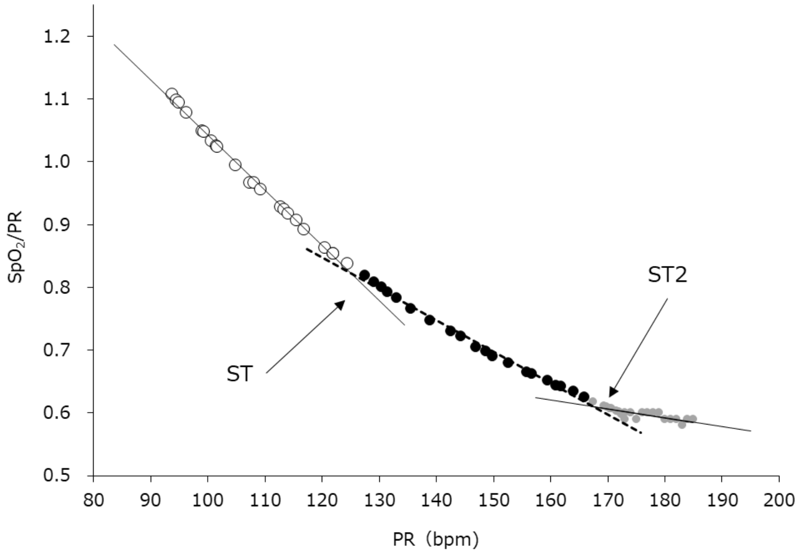

Supplement: Supplementary file 1 [file medsci-13-00192-s001.zip › Supplementary Filer 1,2/Supplementary Filer 1.pdf]
