# Supplementary figures and images for: A Novel Method to Determine the Respiratory Compensation Point from Percutaneous Oxygen Saturation of Healthy Adults During a Ramp-Incremental Test: A Cross-Sectional Study
Source: Med Sci (Basel). 2025 Sep 15;13(3):192. doi: 10.3390/medsci13030192 (PMC12452715; doi:10.3390/medsci13030192)

S2. The correlation plot between ST2 and each Vt2

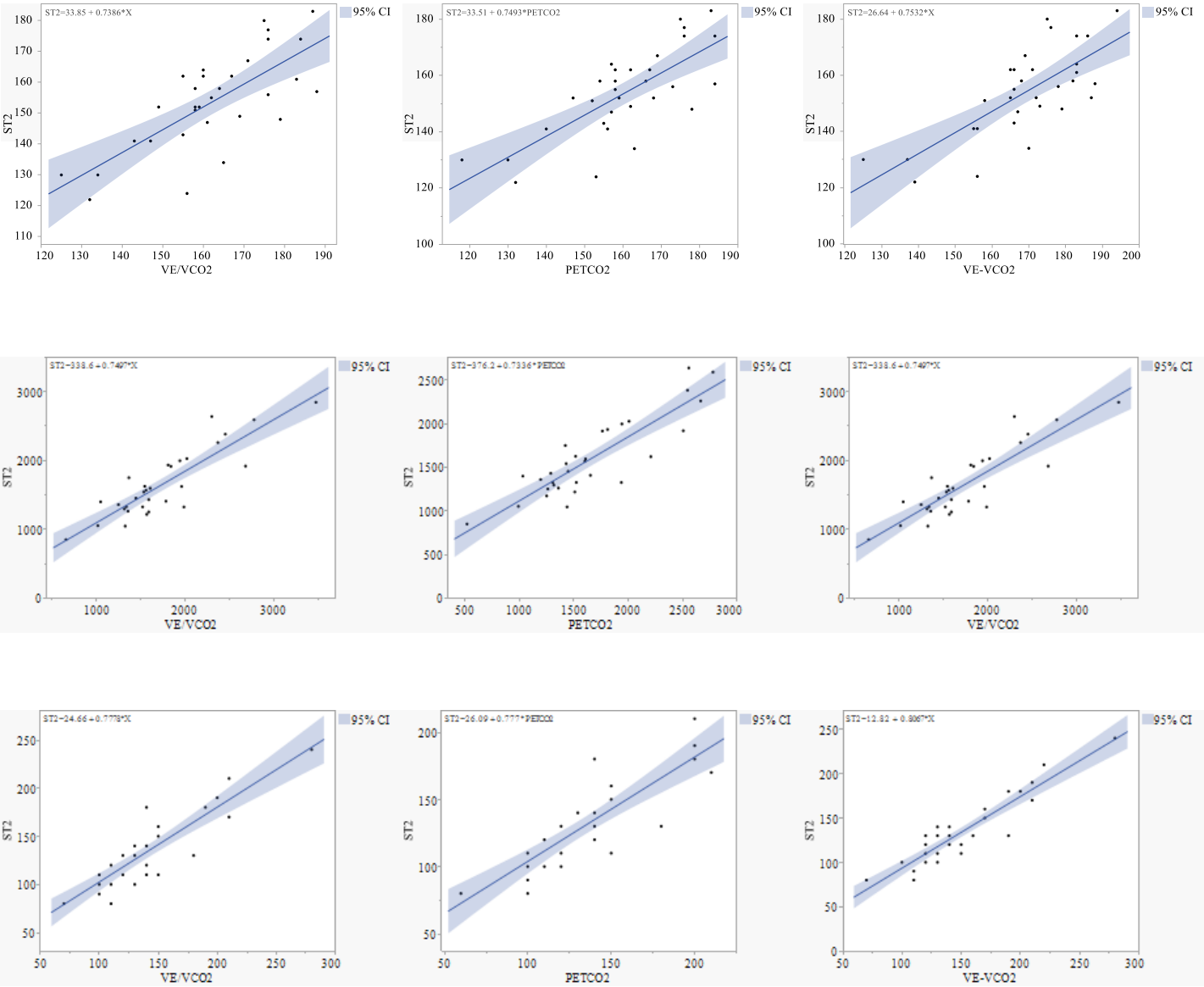

Supplement: Supplementary file 1 [file medsci-13-00192-s001.zip › Supplementary Filer 1,2/Supplementary Filer 2.pdf]
